# Patterns and determinants of drug–drug interactions among community-dwelling older adults in Saudi Arabia: a cross-sectional study

**DOI:** 10.3389/fphar.2026.1830900

**Published:** 2026-05-13

**Authors:** Wael Y. Khawagi, Jehad A. Aldali, Reem M. Aljohani, Abdullah A. Alshehri

**Affiliations:** 1 Department of Clinical Pharmacy, College of Pharmacy, Taif University, Taif, Saudi Arabia; 2 Department of Pathology, College of Medicine, Imam Mohammad Ibn Saud Islamic University (IMSIU), Riyadh, Saudi Arabia; 3 Pharmaceutical Care Department, King Abdulaziz Medical City - WR, Ministry of National Guard–Health Affairs, Jeddah, Saudi Arabia

**Keywords:** chronic disease, drug–drug interactions, geriatric pharmacotherapy, medication safety, older adults, outpatient care, polypharmacy, Saudi Arabia

## Abstract

**Background:**

Polypharmacy is increasingly common among older adults due to the high prevalence of chronic conditions, contributing to a greater risk of potential drug–drug interactions (DDIs). However, limited evidence exists on the patterns and risk factors of DDIs in community-dwelling older adults in Saudi Arabia. This study investigated the patterns, and determinants of DDIs among older outpatients.

**Methods:**

A cross-sectional study was conducted between November 2024 and March 2025, targeting patients aged ≥60 years. Data were collected through structured face-to-face interviews. Potential DDIs were identified using Stockley’s Drug Interactions database. Descriptive statistics were used to characterize DDI prevalence, severity, and drug pairs. A *post hoc* review assessed the likely clinical actions and management recommendations associated with the most frequent DDI pairs. Multivariate logistic regression was used to determine predictors of overall and severe DDIs.

**Results:**

A total of 285 potential DDIs were identified among 173 participants, with 67.2% experiencing at least one interaction. Potential DDIs classified as moderate and severe accounted for 69.1% and 23.2% of cases, respectively. Amlodipine, aspirin, and insulin were the most commonly implicated medications, while atorvastatin, perindopril, and gliclazide were frequently involved in severe interactions. Polypharmacy (≥5 medications) was significantly associated with both overall (aOR = 10.50; 95% CI: 2.02–54.64) and potential severe DDIs (aOR = 14.33; 95% CI: 3.84–53.55). Hypertension was significantly associated with an increased likelihood of overall potential DDIs (aOR = 4.81; 95% CI: 2.10–11.03) and potential severe DDIs (aOR = 6.81; 95% CI: 2.25–20.66).

**Conclusion:**

Potential DDIs are common among older outpatients in Saudi Arabia, particularly among those with hypertension and higher medication burden. Although many interactions were classified as moderate or severe, most required monitoring rather than therapy modification, and their clinical relevance may vary depending on dosage and treatment context. Routine DDI screening and pharmacist-led medication review may enhance medication safety.

## 1 Introduction

The burden of chronic diseases and polypharmacy among older adults is rising globally as populations continue to age (Dagli and Sharma, 2014). According to the World Health Organization, by 2050, the global population aged 60 years and older is expected to reach 2 billion, nearly doubling from 2020 (World Health Organization, 2022). This demographic shift is accompanied by a growing prevalence of age-related chronic conditions such as hypertension, diabetes mellitus, cardiovascular disease, and osteoarthritis, necessitating long-term pharmacological treatment (Corsonello et al., 2010; Luu and Palczewski, 2018).

In Saudi Arabia, the elderly population is expanding rapidly due to improvements in healthcare access and increased life expectancy (Salam, 2023). With aging comes a greater reliance on medications to manage multiple chronic diseases (Du et al., 2020). Recent studies from Saudi Arabia have highlighted a substantial burden of potentially inappropriate prescribing and medication-related risks among older adults (Prabahar, 2025; Prabahar et al., 2024). Consequently, polypharmacy has become increasingly prevalent among older adults (Midão et al., 2018). While the use of multiple drugs may be clinically appropriate, it also introduces a heightened risk of drug–drug interactions (DDIs), which can compromise patient safety and treatment outcomes (Hermann et al., 2021). DDIs are a significant cause of adverse drug events (ADEs) and are associated with increased rates of emergency department visits, hospital admissions, and healthcare costs (Rodrigues and de Oliveira, 2016; AL‐Musawe et al., 2020). These interactions may lead to diminished therapeutic effects, drug toxicity, or unanticipated side effects (Pichini et al., 2023). In older adults, the risks are further amplified due to age-related physiological changes affecting drug absorption, distribution, metabolism, and excretion (Hämmerlein et al., 1998; Maher et al., 2021). Additionally, older adults often experience reduced renal and hepatic function, which can alter drug clearance and increase susceptibility to harmful interactions (Soejima et al., 2022). Managing DDIs in clinical practice, particularly in outpatient settings, remains challenging because, unlike hospitalized patients who receive regular medication reviews, older adult outpatients often see multiple healthcare providers, increasing the risk of fragmented care and suboptimal medication management (Aparasu et al., 2007; Wannawichate and Limpawattana, 2023). Regular DDI screening and medication reconciliation are essential but are not always systematically implemented in routine outpatient care (Peabody et al., 2018).

Despite the clinical importance of DDIs, there is a paucity of real-world data from Saudi Arabia examining their prevalence, severity, and predictors in community-dwelling older adults (Hughes et al., 2023). Existing research has predominantly focused on hospitalized populations or lacks comprehensive interaction assessments using standardized tools (Valuck et al., 2000; Zheng et al., 2018). Moreover, previous studies have rarely stratified interactions by severity or explored which medications are most implicated, particularly those that may lead to severe outcomes requiring clinical intervention (Thillainadesan et al., 2018; Shetty et al., 2018). To address these gaps, this study aimed to evaluate the prevalence and severity of potential DDIs among older patients attending outpatient clinics in Taif, a major city in western Saudi Arabia with demographic and healthcare characteristics similar to national trends. Its role as a regional referral center and its growing elderly population make it a representative setting for studying outpatient prescribing patterns and DDI risks among older adults.

## 2 Methods

### 2.1 Study design and setting

This was a cross-sectional study conducted among older adults patients (≥60 years) who visited outpatient clinics in Taif, Saudi Arabia. The dataset collected between November 2024 and March 2025, was obtained from a prior study on herbal and dietary supplement use (Khawagi et al., 2025). That publication examined supplement users to characterize patterns of supplement intake and potential drug–supplement interactions. However, the present study focuses exclusively on prescription medications to identify and evaluate DDIs. Only participants taking two or more prescribed medications were included, forming a narrower and analytically distinct subset of the original dataset. The current analysis addresses a different research question and reports distinct outcomes, with no overlap in outcomes, analyses, tables, or reported results with the prior publication. Accordingly, this work represents a separate and justified secondary analysis rather than fragmented or duplicate publication.

### 2.2 Data source and participant selection

Data were collected through face-to-face structured interviews conducted by trained pharmacy interns serving as research assistants, under the supervision of licensed pharmacists and academic faculty. The survey was completed electronically during the interviews using the SurveyMonkey platform to ensure accurate and secure data collection. To ensure the accuracy of medication information, participants were asked to present their current medication lists, prescriptions, or medication containers during the interview whenever possible, allowing verification of drug names and dosages. The questionnaire was developed based on existing literature and reviewed by a clinical academic pharmacist for content validity (Agbabiaka et al., 2018; Jermi et al., 2019). All participants provided informed consent, which included permission for future research use of their data. The original questionnaire consisted of 17 items divided into four sections: (1) sociodemographic characteristics covering age, sex, nationality, education level, marital status, and employment status; (2) dietary supplement use assessing types, frequency, dosage, source of recommendation, and place of purchase; (3) side effects and reactions documenting any adverse events related to supplement use; and (4) prescription medication use collecting information on prescribed drugs to evaluate potential drug–supplement interactions. For the current analysis, only data related to sociodemographic characteristics and prescription medication use were extracted to assess potential DDIs among older adults. Participants were recruited using a non-probability convenience sampling technique from outpatient clinics. The inclusion criteria were older adults aged ≥60 years, using at least one prescription medication, who visited outpatient clinics in Taif. Exclusion criteria included individuals unwell to complete the interview and those unable to provide complete medication information. For this analysis, only participants who reported concurrent use of at least two prescription medications were included to enable meaningful assessment of potential DDIs.

### 2.3 Pilot testing

The original questionnaire was pilot tested with 10 participants from the target population to ensure clarity and relevance. Feedback from the pilot study informed minor wording adjustments. Data from pilot participants were excluded from final analysis.

### 2.4 Sample size calculation

The sample size was initially calculated using the Raosoft online calculator, assuming a 95% confidence level, 5% margin of error, and 50% response distribution, which yielded a target of 377 participants for prevalence estimation. However, this study was based on a secondary analysis of an available dataset, and the final analytic sample included 173 eligible older adults. Although this sample was smaller than the original target for prevalence estimation, the adequacy of the multivariable logistic regression analyses was evaluated based on the event-per-variable (EPV) principle. The number of outcome events available for the primary and secondary analyses was considered sufficient to support the planned regression models.

### 2.5 Identification of potential drug–drug interactions

Potential DDIs were assessed by three licensed clinical pharmacists. Participants’ reported prescription medications were systematically reviewed using Stockley’s Drug Interactions (Baxter and Preston, 2019), a widely recognized and evidence-based reference for evaluating the clinical significance of DDIs. Each possible drug pair was screened, and any identified interaction was classified according to Stockley’s criteria as minor, moderate, or severe, based on the potential for clinical impact and the need for intervention.

### 2.6 Statistical analysis

Descriptive statistics were used to summarize participants’ sociodemographic and clinical characteristics, medication profiles, and the prevalence and severity of potential DDIs. Categorical variables were reported as frequencies and percentages, while continuous variables were summarized using means and standard deviations.

Two separate multivariate logistic regression analyses were conducted to examine factors associated with (1) having at least one potential DDI, and (2) having at least one potential DDI classified as severe, based on severity classification from Stockley’s Drug Interactions database. Predictor variables included age group, sex, number of prescription medications, presence of chronic conditions, and other relevant characteristics. Polypharmacy was defined as the use of ≥5 medications. For regression analyses, the number of medications was modeled as a categorical variable (2, 3, 4, and ≥5 medications) to allow assessment of a graded relationship between medication count and the risk of DDIs. Multicollinearity was assessed using variance inflation factors (VIFs), and no evidence of problematic collinearity was observed (all VIFs <2). Model fit was evaluated using the Hosmer–Lemeshow goodness-of-fit test, which indicated acceptable fit for both models (p = 0.780 for overall potential DDIs and p = 0.832 for potential DDI classified as severe).

To minimize overfitting, the regression models were constructed using a parsimonious approach, with the number of predictors constrained relative to the number of outcome events. Categories with relatively small sample sizes (e.g., ≥5 medications) were retained due to their clinical importance; however, the resulting estimates were interpreted with caution.

Results were reported as adjusted odds ratios (aORs) with 95% confidence intervals (CIs). Statistical significance was set at p < 0.05. All analyses were performed using Stata version 16 (StataCorp LLC, College Station, TX, United States of America).

A *post hoc* descriptive analysis was performed on the most frequently reported potential DDI pairs to assess their likely clinical relevance. This assessment used severity classifications and recommended actions from Stockley’s Drug Interactions and included interpretation of expected real-world significance based on standard clinical practice.

### 2.7 Ethical considerations

The study received ethical approval from the Scientific Research Ethics Committee at Taif University, Saudi Arabia (Approval No.: 46-070; approved on 3 November 2024) and the Scientific Research Ethics Committee at King Faisal Medical Complex, Taif (TAIF Health Cluster), Saudi Arabia (Approval No.: 2024-E-94; approved on 28 December 2024). The study was conducted in accordance with the ethical principles of the Declaration of Helsinki and its subsequent amendments. Written informed consent was obtained from all participants prior to study participation, including permission for the use of anonymized data in future research. All data were de-identified prior to analysis to ensure participant confidentiality and privacy.

## 3 Results

### 3.1 Sociodemographic characteristics of participants

A total of 173 participants were included in the study (Table 1). More than half were female (55.4%), and the vast majority were Saudi nationals (97.1%). Nearly half of the participants (46.8%) had no formal education, and most were married (73.4%). The predominant age groups were 60–69 years, representing over half of the sample. Chronic conditions were common, particularly diabetes (71.6%) and hypertension (67.6%), followed by cardiovascular disease (31.7%). Regarding medication use, the total number of prescribed medications was 575, with the majority of participants taking two or three medications.

**TABLE 1:** Sociodemographic and clinical characteristics of the study participants (N = 173).

Characteristic	n (%)
Gender	
Female	96 (55.4%)
Male	77 (44.5%)
Nationality	
Saudi	168 (97.1%)
Non-Saudi	5 (2.9%)
Education	
No formal	81 (46.8%)
Primary	30 (17.3%)
Intermediate	18 (10.4%)
Secondary	17 (9.8%)
Diploma/vocational	6 (3.5%)
Bachelor’s degree or above	21 (12.2%)
Marital status	
Married	127 (73.4%)
Widowed	41 (23.7%)
Divorced	5 (2.9%)
Age group	
60–64	46 (26.6%)
65–69	51 (29.5%)
70–74	42 (24.3%)
75–79	16 (9.2%)
80+	18 (10.4%)
Condition	
Hypertension	117 (67.6%)
Diabetes	124 (71.6%)
CVD	55 (31.7%)
CKD	7 (4.0%)
Asthma	8 (4.6%)
Arthritis	10 (5.7%)
Osteoporosis	9 (5.2%)
Number of medications	
2	66 (38.2%)
3	53 (30.6%)
4	30 (17.3%)
5	9 (5.2%)
6	11 (6.4%)
7	4 (2.3%)

Abbreviations: CVD, cardiovascular disease; CKD, chronic kidney disease.

### 3.2 Frequency and severity of potential drug–drug interactions

Among the 173 participants who reported taking prescription medications, a total of 285 potential DDIs were identified. DDIs were observed in 117 participants, representing 67.2% of the sample, while 57 participants (32.8%) had no identified interactions. Most participants experienced either one (30.1%) or two (14.5%) interactions, with smaller proportions reporting three (7.5%) or four (6.4%) interactions. Approximately 9.2% of participants experienced between five and eleven interactions. In terms of severity, potential DDIs classified as moderate were the most prevalent, affecting 69.1% of the potential interactions. Potential DDIs classified as severe accounted for 23.2% of all identified interactions, including two individuals who had three severe DDIs each. Mild interactions were less common, reported in 7.7% of the sample.

### 3.3 Most frequently involved medications in drug–drug Interaction

**FIGURE 1:**
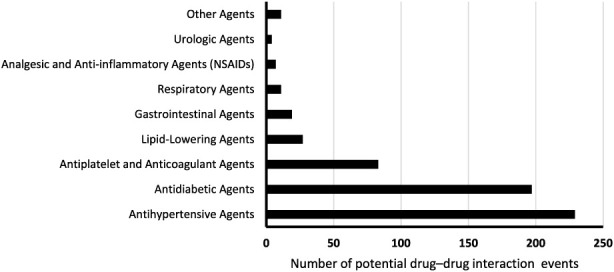
Frequency of medication classes involved in potential drug–drug interaction events among study participants.

Among all recorded DDI events, certain medications were involved more frequently than others. Amlodipine was the most commonly implicated drug, appearing in 103 interactions, followed by aspirin (n = 74), insulin (n = 55), and metformin (n = 49). Other frequently involved medications included perindopril (n = 36), gliclazide (n = 34), and empagliflozin (n = 29). The most frequently involved medications classes in DDI events are summarized in Figure 1. Beyond overall frequency, we also examined the number of unique DDI combinations for each medication. This metric reflects the breadth of a drug’s interaction profile by identifying how many distinct pairings each drug was involved in, regardless of how often each occurred. Both amlodipine and aspirin were involved in 19 unique DDI pairs, indicating the widest interaction profiles. Insulin was involved in 16 unique combinations, while empagliflozin and indapamide were each implicated in 15.

### 3.4 Most common drug–drug interaction pairs and their clinical significance

The analysis identified several DDI pairs that were reported with higher frequency. The most common interaction was between metformin and amlodipine (n = 26), followed by insulin + amlodipine (n = 18) and atorvastatin + amlodipine (n = 14). Most of the reported interactions were of moderate severity and typically required clinical awareness rather than immediate intervention. Although some interactions were classified as severe—such as atorvastatin + amlodipine, insulin + perindopril, and perindopril + gliclazide—these classifications are based on reference criteria, and their clinical relevance may vary depending on factors such as dosage, treatment context, and patient-specific characteristics.

To further characterize these interactions, a *post hoc* descriptive assessment of the most frequent DDI pairs was conducted using Stockley’s recommended clinical actions (Table 2). This assessment indicated that many interactions classified as moderate or severe were associated with recommendations for monitoring or clinical awareness rather than immediate therapy modification. For example, metformin + amlodipine and insulin + amlodipine was categorized as moderate interactions generally requiring awareness, while atorvastatin + amlodipine, although classified as severe, was typically associated with monitoring recommendations. As dosage data were not collected, dose-dependent variation in clinical relevance could not be assessed. The most frequently reported DDI pairs, along with their severity classifications and recommended actions, are summarized in Table 2, while the full list of identified interactions is provided in the Supplementary File (Supplementary Table S1).

**TABLE 2:** Top reported drug–drug interaction pairs with severity and clinical recommendations.

Frequency	Drug pair	Severity	Action	Likely clinical relevance
26	Metformin + amlodipine	Moderate	No particular precautions normally seem to be necessary. Bear the potential for interaction in mind if an otherwise unexplained worsening of diabetic control occurs	Low – awareness only
18	Insulin + amlodipine	Moderate	No particular precautions normally seem to be necessary. Bear the potential for interaction in mind if an otherwise unexplained worsening of diabetic control occurs	Low – awareness only
14	Atorvastatin + amlodipine	Severe	Bear an interaction in mind in the case of increased atorvastatin adverse effects	Moderate – dose-dependent risk
11	Gliclazide + amlodipine	Moderate	No particular precautions normally seem to be necessary. Bear the potential for interaction in mind if an otherwise unexplained worsening of diabetic control occurs	Low – awareness only
11	Aspirin + insulin	Moderate	Be aware that large doses of salicylates might affect blood glucose concentration in patients with diabetes and adjust the insulin dose accordingly	Low–moderate – depends on aspirin dose
10	Metformin + aspirin	Moderate	Be aware that large doses of salicylates might affect blood glucose concentration in patients with diabetes and adjust the insulin dose accordingly	Low–moderate – depends on aspirin dose
9	Insulin + perindopril	Severe	Warn patients newly starting an ACE inhibitor that excessive hypoglycaemia has rarely been seen. Any problem seems easily resolved by reducing the insulin dose	Moderate – requires monitoring
9	Perindopril + aspirin	Moderate	Hypertension: no action needed with low-dose aspirin. For high-dose aspirin suspect an interaction if blood pressure control is erratic: increase ACE inhibitor dose or consider an alternative analgesics. Heart failure: avoid aspirin unless specifically indicated (e.g. ischaemic disease)	Low–moderate – depends on indication/dose
8	Amlodipine + aspirin	Moderate	Bear in mind the potential for an interaction if blood pressure control is difficult in patients taking analgesic dose aspirin or if unexplained bleeding occurs	Low – usually no action at low-dose aspirin
7	Aspirin + bisoprolol fumarate	Moderate	Only some patients are affected. Consider monitoring blood pressure if an NSAID is started or stopped. Note that NSAIDs should generally be avoided in those with heart failure	Low – depends on NSAID use
6	Perindopril + gliclazide	Severe	Warn patients newly starting an ACE inhibitor that excessive hypoglycaemia has rarely been seen. Any problem seems easily resolved by reducing the sulfonylurea dose	Moderate – requires monitoring
6	Valsartan + aspirin	Moderate	No action needed if low-dose aspirin is used. Suspect an interaction with high-dose aspirin if the angiotensin-II receptor antagonist seems less effective or blood pressure control is erratic. Consider an alternative analgesic	Low – no action at low-dose aspirin

### 3.5 Factors associated with potential drug–drug interactions

Multivariable logistic regression analysis was conducted to examine factors associated with potential DDIs among participants taking prescription medications (Table 3). After adjusting for all included variables, male participants had higher odds of experiencing a DDI compared to females (aOR = 2.27, 95% CI: 0.98–5.29), though this association was marginal (p = 0.057). Hypertension was significantly associated with an increased likelihood of potential DDIs (aOR = 4.81; 95% CI: 2.10–11.03; p < 0.001). Diabetes was also associated with increased odds of DDIs (aOR = 2.91, 95% CI: 1.28–6.61, p = 0.011).

**TABLE 3:** Multivariate logistic regression analysis of factors associated with the presence of potential drug–drug interactions.

Factors	DDI prevalence (%)	Adjusted odds ratio (aOR)	95% CI	p-value
Gender				
Female (ref)	62.11	1.00	—	—
Male	73.08	2.27	0.98–5.29	0.057
Hypertension				
No (ref)	41.07	1.00	—	—
Yes	79.66	4.81	2.10–11.03	<0.001
Diabetes	​	​	​	​
No (ref)	44.00	1.00	—	—
Yes	76.61	2.91	1.28–6.61	0.011
Age category				
60–64 (ref)	56.52	1.00	—	—
65–69	65.38	0.80	0.28–2.32	0.685
70–74	71.43	0.88	0.28–2.71	0.818
75–79	81.25	0.96	0.18–5.20	0.965
80+	77.78	1.39	0.30–6.43	0.671
Number of medications				
2 (ref)	43.94	1.00	—	—
3	74.07	3.02	1.26–7.22	0.013
4	86.67	5.84	1.67–20.47	0.006
5+	91.67	10.50	2.02–54.64	0.005

Age category was not significantly associated with DDIs in the adjusted model, with all age groups showing non-significant differences compared to the reference group (60–64 years). In contrast, polypharmacy remained significantly associated with an increased likelihood of potential DDIs. Compared to participants taking two medications, those taking three had significantly higher odds of experiencing a DDI (aOR = 3.02, 95% CI: 1.26–7.22, p = 0.013), with risk further increasing for those taking four (aOR = 5.84, p = 0.006) or five or more medications (aOR = 10.50, p = 0.005).

### 3.6 Factors associated with of potential DDIs classified as severe

Table 4 presents the results of the multivariate logistic regression assessing factors associated with of potential DDIs classified as severe. Although male participants had higher adjusted odds of experiencing potential DDIs classified as severe than females (aOR = 2.30, 95% CI: 0.95–5.54), this association did not reach statistical significance (p = 0.064). Hypertension showed the strongest association with potential DDIs classified as severe. Participants with hypertension had nearly seven times the odds of experiencing a potential DDIs classified as severe compared to those without (aOR = 6.81, 95% CI: 2.25–20.66, p < 0.001). Diabetes was not significantly associated with potential DDIs classified as severe in the adjusted model (aOR = 0.66, p = 0.401). Age group was not a significant factors of potential DDIs classified as severe, with all age categories showing non-significant differences relative to the reference group. However, the number of medications was strongly associated with potential DDIs classified as severe. Compared to those taking two medications, participants taking three had an aOR of 3.28 (p = 0.031), those taking four had an aOR of 17.79 (p < 0.001), and those on five or more had an aOR of 14.33 (p < 0.001).

**TABLE 4:** Multivariate logistic regression analysis of factors associated with the presence of potential DDIs classified as severe.

Factor	Severe DDI prevalence (%)	Adjusted odds ratio (aOR)	95% CI	p-value
Gender	​	​	​	​
Female (ref)	28.42	1	-	-
Male	33.33	2.3	0.95–5.54	0.064
Hypertension	​	​	​	​
No (ref)	8.93	1	-	-
Yes	40.68	6.81	2.25–20.66	<0.001
Diabetes	​	​	​	​
No (ref)	24	1	-	-
Yes	33.06	0.66	0.25–1.74	0.401
Age category	​	​	​	​
60–64 (ref)	23.91	1	-	-
65–69	26.92	0.86	0.27–2.77	0.806
70–74	35.71	1.05	0.34–3.25	0.935
75–79	43.75	1.09	0.25–4.64	0.912
80+	33.33	1.2	0.28–5.10	0.809
Number of medications	​	​	​	​
2 (ref)	9.09	1	-	-
3	27.78	3.28	1.11–9.66	0.031
4	63.33	17.79	5.17–61.22	<0.001
5+	54.17	14.33	3.84–53.55	<0.001

## 4 Discussion

This study assessed the prevalence, severity, and associated factors of potential DDIs among community-dwelling elderly patients attending outpatient clinics in Taif, Saudi Arabia. The findings reveal that DDIs are highly prevalent in this population, with two-thirds of participants who were taking two or more prescription medications experiencing at least one potential interaction. Notably, about one-third of participants had at least one severe DDI, highlighting a substantial risk of clinically significant harm.

However, it is important to note that potential DDIs, as identified in this study, do not necessarily indicate clinical harm. The actual significance of an interaction depends on factors such as dose, duration, comorbidities, and patient monitoring. To help contextualize these findings, we conducted a descriptive *post hoc* review of the top reported DDI pairs using Stockley’s recommended clinical actions (Table 3). This review showed that many flagged interactions, particularly those categorized as moderate, require only clinical awareness and not therapeutic changes. Even some severe interactions are typically dose-dependent and may not be clinically relevant in all cases. This distinction is critical for interpreting the prevalence and potential risk of DDIs in routine practice.

Our findings are consistent with previous studies reporting moderate-to-high DDI prevalence among older adults, particularly in settings with high rates of polypharmacy (Kaliamurthy et al., 2015). Recent studies from Europe, Asia, and the Middle East have reported similar trends in DDI prevalence ranging from 26% to over 91%, depending on population characteristics and the interaction database used (Hughes et al., 2023; Midão et al., 2018; Alsuwaidan et al., 2019; Alkan et al., 2017; Qerem et al., 2018; Burato et al., 2021; Jiang et al., 2025). The majority of DDIs identified were of moderate severity, which typically require monitoring or clinical awareness rather than urgent intervention. However, a noteworthy proportion of DDIs were classified as severe, potentially necessitating dose adjustment, therapy modification, or close clinical monitoring. The pattern of medications involved in DDIs reflects common prescribing practices for cardiometabolic conditions in older adults (Khawagi et al., 2024; Manirajan and Sivanandy, 2023; Alshehri et al., 2024). However, their clinical relevance may vary depending on context.

Multivariate logistic regression analysis revealed that polypharmacy was the strongest associated factor of both overall and severe DDIs. Compared to those on two medications, participants taking five or more medications had more than a tenfold increase in the odds of having at least one DDI and a fourteenfold increase in the odds of having a severe interaction. These findings underscore the critical role of medication burden in increasing interaction risk (Rasool et al., 2023). Hypertension and diabetes were also significantly associated with an increased likelihood of DDIs (Subramanian et al., 2018). Patients with hypertension were nearly five times more likely to experience any DDI and almost seven times more likely to have a severe DDI (Fravel and Ernst, 2021). This association likely reflects greater treatment complexity and medication burden rather than a direct effect of the condiditon itself, and residual confounding related to treatment intensity and prescribing patterns may persist despite adjustment (Ingersoll and Cohen, 2008). Interestingly, age and gender were not independently associated with DDIs in the adjusted models, suggesting that clinical factors and medication count may be more influential determinants than demographic variables alone (Hughes et al., 2021; Zerah et al., 2021).

These findings highlight the need for proactive medication review and interaction screening as part of routine care for older adults, especially those with chronic diseases and polypharmacy (Sheikh-Taha and Asmar, 2021; Zhao et al., 2022). Integrating clinical pharmacists into outpatient care teams can play a pivotal role in identifying and managing DDIs through medication reconciliation and individualized counselling (Phatak et al., 2016; Al-Hashar et al., 2018; Alqenae et al., 2020). Additionally, developing context-specific DDI screening tools that account for local prescribing patterns could enhance detection and clinical decision-making (Duke and Bolchini, 2011). Detecting potential DDIs in daily practice can be challenging. Therefore, integrating DDI-screening and clinical decision-support tools within electronic prescribing and dispensing systems can assist healthcare professionals by providing real-time alerts and evidence-based recommendations. Incorporating such systems, along with regular medication review and pharmacist involvement, may further enhance medication safety among older adults (Rieckert et al., 2018; Horsky et al., 2013).

Future research should aim to evaluate the clinical outcomes associated with identified DDIs, such as hospitalization or adverse drug events, and assess the effectiveness of targeted interventions in reducing DDI-related harm in elderly populations. Longitudinal studies incorporating actual dosage, timing, and laboratory data would also help refine risk stratification and better determine the clinical relevance of potential interactions.

A key strength of this study is the use of a clinically validated DDI reference alongside assessment by multiple licensed pharmacists, enhancing the reliability of interaction classification. Additionally, the focus on community-dwelling older adults provides valuable real-world insight in the under-researched context of Saudi Arabia.

However, several limitations should be acknowledged. First, the use of a non-probability convenience sampling approach may introduce selection bias and limit the representativeness of the sample. Individuals with greater healthcare engagement or more complex medication regimens may have been more likely to participate, potentially leading to an overrepresentation of patients at higher risk of potential DDIs and inflating the observed prevalence. Second, the analysis was based on self-reported medication use, which may be subject to recall bias. Third, while potential DDIs were identified using a standardized reference, actual adverse outcomes were not assessed, and the clinical relevance of flagged interactions may vary depending on factors such as dosage, treatment duration, comorbidities, and renal function. To partially address this, a descriptive *post hoc* review of the most frequently reported DDIs was conducted using Stockley’s clinical action recommendations to better contextualize their likely relevance in routine care. However, as dosage and timing data were not systematically collected, interactions were assessed at the drug-pair level, which may not fully reflect real-world risk. For example, the interaction between amlodipine and atorvastatin is dose-dependent and may pose minimal risk at lower doses.

Additionally, the sample predominantly included patients with hypertension and diabetes managed according to clinical guidelines, resulting in common and often unavoidable drug combinations with known interaction potential. The analysis focused exclusively on prescription medications; herbal and dietary supplements, although collected, were excluded as they were examined in a separate study (Khawagi et al., 2025). By isolating prescription DDIs and applying severity classification and multivariable modeling, this study provides distinct and clinically relevant insights into medication-related risks and their predictors. Finally, the relatively small sample size and single geographic setting may limit statistical power to detect less common interactions, contribute to wider confidence intervals for some estimates, and reduce the generalizability of the findings to the broader older adult population.

## 5 Conclusion

In conclusion, this study highlights a substantial burden of potential DDIs among community-dwelling elderly outpatients in Saudi Arabia, particularly among those with polypharmacy and chronic conditions such as hypertension and diabetes. Routine DDI screening, particularly for commonly implicated medications, should be prioritized in geriatric care to reduce the risk of preventable drug-related harm. Future studies should incorporate clinical outcomes, laboratory data, and patient-specific dosing to better determine the true impact of DDIs in older adults.

## Data Availability

The data analyzed in this study is subject to the following licenses/restrictions: The datasets generated and analyzed during the current study are not publicly available due to ethical and privacy restrictions related to human participant confidentiality. De-identified data may be made available from the corresponding author upon reasonable request and with permission from the relevant institutional ethics committee. Requests to access these datasets should be directed to AA; a.aalshehri@tu.edu.sa.
