# Supplementary material for: Patterns and determinants of drug–drug interactions among community-dwelling older adults in Saudi Arabia: a cross-sectional study
Source: Front Pharmacol. 2026 May 13;17:1830900. doi: 10.3389/fphar.2026.1830900 (PMC13212434; doi:10.3389/fphar.2026.1830900)
Supplement: Supplementary file 1 [file Table1.pdf]

Table S1: Complete list of reported drug–drug interaction pairs identified in the study, including their frequency, severity classification, and recommended actions, as extracted from Stockley's Drug Interactions

Frequency	Medication	Medication	Severity	Action
**26**	Metformin	Amlodipine	Moderate	No particular precautions normally seem to be necessary. Bear the potential for interaction in mind if an otherwise unexplained worsening of diabetic control occurs.
**18**	Insulin	Amlodipine	Moderate	No particular precautions normally seem to be necessary. Bear the potential for interaction in mind if an otherwise unexplained worsening of diabetic control occurs.
**14**	Atorvastatin	Amlodipine	Severe	Bear an interaction in mind in the case of increased atorvastatin adverse effects.
**11**	Gliclazide	Amlodipine	Moderate	No particular precautions normally seem to be necessary. Bear the potential for interaction in mind if an otherwise unexplained worsening of diabetic control occurs.
**11**	Aspirin	Insulin	Moderate	Be aware that large doses of salicylates might affect blood glucose concentration in patients with diabetes and adjust the insulin dose accordingly.
**10**	Metformin	Aspirin	Moderate	Be aware that large doses of salicylates might affect blood glucose concentration in patients with diabetes and adjust the insulin dose accordingly.
**9**	Insulin	Perindopril	Severe	Warn patients newly starting an ACE inhibitor that excessive hypoglycaemia has rarely been seen. Any problem seems easily resolved by reducing the insulin dose.
**9**	Perindopril	Aspirin	Moderate	Hypertension: no action needed with low-dose aspirin. For high-dose aspirin suspect an interaction if blood pressure control is erratic: increase ACE inhibitor dose or consider an alternative analgesics. Heart failure: avoid aspirin unless specifically indicated (e.g. ischaemic disease).
**8**	amlodipine	Aspirin	Moderate	Bear in mind the potential for an interaction if blood pressure control is difficult in patients taking analgesic dose aspirin or if unexplained bleeding occurs.
**7**	Aspirin	Bisoprolol fumarate	Moderate	Only some patients are affected. Consider monitoring blood pressure if an NSAID is started or stopped. Note that NSAIDs should generally be avoided in those with heart failure
**6**	Perindopril	Gliclazide	Severe	Warn patients newly starting an ACE inhibitor that excessive hypoglycaemia has rarely been seen. Any problem seems easily resolved by reducing the sulfonylurea dose.
**6**	Valsartan	Aspirin	Moderate	No action needed if low-dose aspirin is used. Suspect an interaction with high-dose aspirin if the angiotensin-II receptor antagonist seem less effective or blood pressure control is erratic. Consider an alternative analgesic
**4**	Metformin	indapamide	Moderate	If higher doses are used, increased monitoring of diabetic control would seem prudent.
**4**	Asprin	Gliclazide	Moderate	Be aware that large doses of salicylates might affect blood glucose concentrations in patients with diabetes and adjust the antidiabetic dose accordingly.
**4**	empagliflozin	gliclazide	Moderate	Diabetic control should be monitored when any new antidiabetic drug is added and dose adjustments should be made as needed.
**4**	Bisoprolol	Insulin	Severe	Monitor concurrent use and adjust the insulin dose as necessary. Cardioselective beta blockers (such as bisoprolol) are less likely to interact. Warn patients about the possible absence of hypoglycaemic warning symptoms (such as tremor, tachycardia).
**4**	Empagliflozin	Perindopril	Moderate	Bear the potential of an increased risk of hypotension in mind. If dizziness, lightheadedness, etc. occur, advise patients to lie down, elevate the legs until dizziness subsides, and get up slowly.
**4**	Bisoprolol	Amlodipine	Mild	No action needed unless an undesirably large decrease in blood pressure occurs
**3**	Indapamide	Valsartan	Moderate	Concurrent use is generally well tolerated and can be clinically beneficial. Monitor blood pressure and potassium levels initially. In patients with heart failure or those who are volume or sodium depleted, the dose of diuretic or angiotensin II receptor antagonist may need to be reduced
**3**	amlodipine	indapamide	Mild	No action needed unless the blood-pressure lowering effects are excessive.
**3**	amlodipine	sitagliptin	Moderate	No particular precautions normally seem to be necessary. Bear the potential for interaction in mind if an otherwise unexplained worsening of diabetic control occurs.
**3**	Atorvastatin	omeprazole	Severe	Bear an interaction in mind in the case of an unexpected response to treatment.
**3**	Pantoprazole	metformin	Mild	No specific dose adjustments needed, but it is prudent to monitor blood glucose concentrations on stopping or starting pantoprazole, and also when any change is made to the medication regimen of a patient with diabetes.
**3**	empagliflozin	atrovastatin	Moderate	Bear an interaction in mind in the case of an unexpected response to treatment.
**3**	empagliflozin	Amlodipine	Moderate	No particular precautions normally seem to be necessary. Bear the potential for interaction in mind if an otherwise unexplained worsening of diabetic control occurs.
**3**	valsartan	spironolacto ne	Severe	Use lowest possible doses of both drugs (UK); the spironolactone dose should not exceed 25 mg daily. Monitor potassium and renal function closely; concurrent use should be stopped, or interrupted, if hyperkalaemia occurs. Angiotensin II receptor antagonists should not be used with spironolactone in patients with marked renal impairment.
**3**	Aspirin	spironolacto ne	Moderate	Consider an interaction if the diuretic response to spironolactone is less than expected in patients taking aspirin. The isolated case of gynaecomastia seems unlikely to be of general relevance.
**3**	empagliflozin	Aspirin	Moderate	Be aware that large doses of salicylates might affect blood glucose concentrations in patients with diabetes and adjust the antidiabetic dose accordingly.
**2**	Carvedilol	Amlodipine	Mild	No action needed unless an undesirably large decrease in blood pressure occurs.
**2**	Indapamide	Aspirin	Mild	If concurrent use is essential monitor renal function, electrolytes, and disease control.
**2**	Insulin	Dulaglutide	Moderate	Diabetic control should be monitored when any new antidiabetic drug is added. Consider reducing the dose of insulin.
**2**	Metformin	Linagliptin	Moderate	No dose adjustment of either drug is needed. Diabetic control should be monitored when any new antidiabetic drug is added and dose adjustments should be made as needed.

**2**	formoterol	budesonide	Severe	The CSM in the UK advises monitoring in severe asthma, because of the probability of multiple potassium-depleting drugs being used, and because of predisposing conditions. Consider monitoring based on the severity of the patients' condition, and the number of potassium-depleting drugs used.
**2**	esomeprazole	atrovastatin	Severe	Bear an interaction in mind in the case of an unexpected response to treatment.
**2**	glipizide	Amlodipine	Moderate	No particular precautions normally seem to be necessary. Bear the potential for interaction in mind if an otherwise unexplained worsening of diabetic control occurs.
**2**	tamsulosin	Amlodipine	Severe	Patients should lie down if dizziness, fatigue or sweating develop and remain lying down until symptoms abate. Patients already taking a calcium-channel blocker should have their dose reduced and begin with a low-dose of alpha blockers, with the first dose taken just before bed.
**2**	Carvedilol	Aspirin	Moderate	Only some patients are affected. Consider monitoring blood pressure if an NSAID is started or stopped. Note that NSAIDs should generally be avoided in those with heart failure.
**2**	warfarin	metformin	Severe	The general significance of this interaction is unknown, but it seems unlikely to be of general importance.
**2**	naproxen	Gliclazide	Moderate	Consider the possibility of an interaction if any unexplained loss of diabetic control occurs on concurrent use.
**2**	Empagliflozin	sitagliptin	Moderate	Diabetic control should be monitored when any new antidiabetic drug is added and dose adjustments should be made as needed.
**2**	Perindopril	sitagliptin	Severe	Be alert to the possibility of an increased risk of angioedema.
**2**	levothyroxine	esomeprazole	Moderate	If an interaction is suspected, monitor thyroid function and adjust the levothyroxine dose accordingly or consider using an oral solution formulation. Any interaction may take several months to develop.
**2**	empagliflozin	furosemide	Moderate	Monitor concurrent use for excessive fluid and electrolyte loss, and hypotension.
**2**	Aspirin	furosemide	Mild	Antiplatelet dose aspirin: bear the possibility of an interaction in mind should a reduced diuretic response occur. Analgesic dose aspirin: avoid where possible. Consider using an alternative non-NSAID analgesic. If concurrent use is necessary, monitor diuretic response, renal function and hearing.
**2**	Valsartan	furosemide	Moderate	Concurrent use is generally well tolerated and can be clinically beneficial. Monitor blood pressure and potassium levels initially. In patients with heart failure or those who are volume or sodium depleted, the dose of diuretic or angiotensin II receptor antagonist may need to be reduced.
**1**	Cortcosteroid	Ventolin inhalation	Severe	Consider monitoring based on the severity of the patients' condition, and the number of potassium-depleting drugs used.
**1**	Insulin	Hydrochlorothiazide	Moderate	If higher doses are used, increased monitoring of diabetic control would seem prudent.
**1**	levothyroxine	omeprazole	Moderate	If an interaction is suspected, monitor thyroid function and adjust the levothyroxine dose accordingly or consider using an oral solution formulation. Any interaction may take several months to develop.
**1**	Perindopril	Tacrolimus Ointment	Moderate	Renal function and potassium concentrations should be closely monitored.
**1**	latanoprost	bimatoprost	Moderate	Concurrent use is not recommended (UK, US). If both drugs are used, the UK manufacturer of bimatoprost advises monitoring intraocular pressure.
**1**	Atenolol	Amlodipine	Mild	No action needed unless an undesirably large decrease in blood pressure occurs.
**1**	amlodipine	chlorothiazide	Mild	No action needed unless the blood-pressure lowering effects are excessive.
**1**	Perindopril	chlorothiazide	Moderate	Start the ACE inhibitor at the lowest dose. Advise patients to lie down if dizziness, lightheadedness, etc, occurs. Furosemide 80 mg daily or more (or equivalent): monitor closely, consider stopping the diuretic 24 hours before starting the ACE inhibitor, or monitor for 2 hours or until the blood pressure is stable.
**1**	Insulin	Linagliptin	Moderate	It is prudent to monitor blood glucose concentrations when any change is made to the medication regimen of a patient with diabetes. An insulin dose reduction should be considered to reduce the risk of hypoglycaemia.
**1**	Insulin	sitagliptin	Moderate	It is prudent to monitor blood glucose concentrations when any change is made to the medication regimen of a patient with diabetes. An insulin dose reduction should be considered to reduce the risk of hypoglycaemia.
**1**	Atorvastatin	imatinib	Severe	If concurrent use is unavoidable, start the statin at the lowest possible dose. In patients already taking statins, the dose of the statin might need to be considerably reduced if imatinib is started. Counsel patients regarding myopathy (e.g. report any unexplained muscle pain, tenderness or weakness), and stop the statin immediately if myopathy does occur.
**1**	diazepam	diclofenac	Moderate	The clinical importance of this interaction is unclear.
**1**	Insulin	propanolol	Severe	Monitor concurrent use and adjust the insulin dose as necessary. Consider changing to a more cardioselective beta blocker (such as atenolol, bisoprolol) as they are less likely to interact. Warn patients about the possible absence of hypoglycaemic warning symptoms (such as tremor, tachycardia).
**1**	Insulin	Pioglitazone	Severe	If oedema or worsening heart failure occurs the causes should be assessed: consider reducing the dose or stopping pioglitazone. To manage any hypoglycaemia the required dose of insulin might need to be reduced.
**1**	amlodipine	Pioglitazone	Moderate	No particular precautions normally seem to be necessary. Bear the potential for interaction in mind if an otherwise unexplained worsening of diabetic control occurs.
**1**	Insulin	indapamide	Moderate	If higher doses are used, increased monitoring of diabetic control would seem prudent.
**1**	Insulin	nifedipine	Moderate	The general importance is uncertain. Bear the potential for interaction in mind if an otherwise unexplained worsening of diabetic control occurs.
**1**	nifedipine	indapamide	Mild	No action needed unless the blood-pressure lowering effects are excessive.
**1**	Perindopril	Isosorbide Dinitrate	Moderate	This seems likely to be a desirable interaction unless the antihypertensive effects are excessive in which case consider reducing the dose of one or both drugs.
**1**	Indapamide	omeprazole	Moderate	Consider monitoring magnesium concentrations before and during treatment if a proton pump inhibitor is used long-term with diuretics.

**1**	tamsulosin	indapamide	Moderate	Patients should lie down if dizziness, fatigue or sweating develop and remain lying down until symptoms abate. A dose reduction and then re-titration may be necessary, particularly for those with congestive heart failure.
**1**	Clopidogrel	Aspirin	Moderate	Advise patients to report any unusual or excessive bleeding. The UK manufacturer advises a maximum dose of aspirin 100 mg daily on the concurrent use of clopidogrel.
**1**	Atorvastatin	pantoprazole	Severe	Bear an interaction in mind in the case of an unexpected response to treatment.
**1**	Clopidogrel	omeprazole	Moderate	Use only where the risk of gastrointestinal bleeding outweighs the risk of clopidogrel treatment failure. Consider giving an H2-receptor antagonist (not cimetidine) or giving dexlansoprazole, rabeprazole, or lower doses of pantoprazole or lansoprazole.
**1**	Pantoprazole	indapamide	Moderate	Consider monitoring magnesium concentrations before and during treatment if a proton pump inhibitor is used long-term with diuretics.
**1**	Indapamide	Meloxicam	Mild	Bear this interaction in mind if blood pressure control is reduced or critical. Heart failure: consider an alternative non-NSAID analgesic. If concurrent use is essential, monitor renal function, electrolytes and disease control: consider increasing the thiazide dose, using an intravenous diuretic or additional diuretic.
**1**	dapagliflozin	Insulin	Moderate	Diabetic control should be monitored when any new antidiabetic drug is added and dose adjustments should be made as needed.
**1**	dapagliflozin	lisinopril	Moderate	Bear the potential of an increased risk of hypotension in mind. If dizziness, lightheadedness, etc. occur, advise patients to lie down, elevate the legs until dizziness subsides, and get up slowly.
**1**	Insulin	lisinopril	Severe	Warn patients newly starting an ACE inhibitor that excessive hypoglycaemia has rarely been seen. Any problem seems easily resolved by reducing the insulin dose.
**1**	empagliflozin	Insulin	Moderate	Diabetic control should be monitored when any new antidiabetic drug is added and dose adjustments should be made as needed.
**1**	empagliflozin	glipizide	Moderate	Diabetic control should be monitored when any new antidiabetic drug is added and dose adjustments should be made as needed.
**1**	warfarin	ranitidine	Severe	This interaction seems rare, but bear it in mind in case of an excessive response to anticoagulant treatment.
**1**	Indapamide	sitagliptin	Moderate	If higher doses are used, increased monitoring of diabetic control would seem prudent.
**1**	Atorvastatin	sitagliptin	Severe	The clinical relevance of the isolated cases is unclear. Any patient taking a statin should be warned of the risk of rhabdomyolysis and counselled regarding myopathy (e.g. report any unexplained muscle pain, tenderness or weakness).
**1**	Clopidogrel	esomeprazole	Moderate	Use only where the risk of gastrointestinal bleeding outweighs the risk of clopidogrel treatment failure. Consider giving an H2-receptor antagonist (not cimetidine) or giving dexlansoprazole, rabeprazole, or lower doses of pantoprazole or lansoprazole.
**1**	Aspirin	telmisartan	Moderate	No action needed if low-dose aspirin is used. Suspect an interaction with high-dose aspirin if the angiotensin-II receptor antagonist seem less effective or blood pressure control is erratic. Consider an alternative analgesic.
**1**	empagliflozin	glimepiride	Moderate	Diabetic control should be monitored when any new antidiabetic drug is added and dose adjustments should be made as needed.
**1**	Bisoprolol	glimepiride	Severe	Serious hypoglycaemic episodes are rare. Patients newly started on a beta blocker should be warned about the possible absence of hypoglycaemic warning symptoms. If diabetic control is disturbed, adjust the antidiabetic dose.
**1**	Gliclazide	sitagliptin	Moderate	It is prudent to monitor blood glucose concentrations when any change is made to the medication regimen of a patient with diabetes.
**1**	Metformin	sitagliptin	Moderate	No dose adjustment of either drug is needed. Diabetic control should be monitored when any new antidiabetic drug is added and dose adjustments should be made as needed.
**1**	empagliflozin	telmisartan	Moderate	Bear the potential of an increased risk of hypotension in mind. If dizziness, lightheadedness, etc. occur, advise patients to lie down, elevate the legs until dizziness subsides, and get up slowly.
**1**	rosuvastatin	Amlodipine	Mild	No action needed.
**1**	ticagrelor	Aspirin	Moderate	Advise patients to report any unusual or excessive bleeding. The recommended dose of aspirin to be used with ticagrelor is 75 to 150 mg daily (75 to 100 mg US).
**1**	ticagrelor	Bisoprolol	mild	Be aware of a possible increased risk of bradycardia.
**1**	Meloxicam	Aspirin	Severe	Consider gastroprotection (e.g. a proton pump inhibitor). Analgesic dose aspirin should not be used with NSAIDs.
**1**	paracetamol	Aspirin	Moderate	The clinical relevance of this finding is unclear, but it seems unlikely to warrant a change in treatment.
**1**	Meloxicam	Perindopril	Moderate	Only some patients are affected. Monitor blood pressure if an NSAID is started, and consider monitoring urea and electrolytes. Consider intermittent use of NSAIDs as a possible cause if erratic blood pressure control occurs.
**1**	salbutamol	indapamide	Severe	The CSM in the UK advises monitoring in severe asthma, because of the probability of multiple potassium-depleting drugs being used, and because of predisposing conditions. Consider monitoring based on the severity of the patients' condition, and the number of potassium-depleting drugs used.
**1**	salmeterol	indapamide	Severe	The CSM in the UK advises monitoring in severe asthma, because of the probability of multiple potassium-depleting drugs being used, and because of predisposing conditions. Consider monitoring based on the severity of the patients' condition, and the number of potassium-depleting drugs used.
**1**	semaglutide	Insulin	Moderate	Diabetic control should be monitored when any new antidiabetic drug is added. Consider reducing the dose of insulin.
**1**	alfuzosin	Amlodipine	Severe	Patients should lie down if dizziness, fatigue or sweating develop and remain lying down until symptoms abate. Patients already taking a calcium-channel blocker should have their dose reduced and begin with a low-dose of alpha blockers, with the first dose taken just before bed.
**1**	Valsartan	Perindopril	Severe	Concurrent use is not recommended and is contraindicated in patients with renal impairment or diabetic nephropathy. If concurrent use is essential (under specialist supervision), monitor blood pressure, renal function, and electrolyte and fluid balance carefully.
**1**	Valsartan	Amlodipine	Severe	Bear an interaction in mind in the case of increased atorvastatin adverse effects.
**1**	empagliflozin	budesonide	Moderate	It might be prudent to increase monitoring of diabetic control in patients requiring high-dose corticosteroids and consider reducing the dose of the inhaled corticosteroid if possible, or adjusting the dose of the antidiabetic medication as necessary.

**1**	Gliclazide	budesonide	Moderate	It might be prudent to increase monitoring of diabetic control in patients requiring high-dose corticosteroids and consider reducing the dose of the inhaled corticosteroid if possible, or adjusting the dose of the antidiabetic medication as necessary.
**1**	Metformin	budesonide	Moderate	It might be prudent to increase monitoring of diabetic control in patients requiring high-dose corticosteroids and consider reducing the dose of the inhaled corticosteroid if possible, or adjusting the dose of the antidiabetic medication as necessary.
**1**	empagliflozin	Linagliptin	Moderate	Diabetic control should be monitored when any new antidiabetic drug is added and dose adjustments should be made as needed.
**1**	empagliflozin	indapamide	Moderate	Monitor concurrent use for excessive fluid and electrolyte loss, and hypotension. If higher doses are used, increased monitoring of diabetic control would seem prudent
**1**	Indapamide	Gliclazide	Moderate	If higher doses are used, increased monitoring of diabetic control would seem prudent.
**1**	liraglutide	Aspirin	Moderate	Be aware that large doses of salicylates might affect blood glucose concentrations in patients with diabetes and adjust the antidiabetic dose accordingly.
**1**	Perindopril	furosemide	Moderate	Start the ACE inhibitor at the lowest dose. Advise patients to lie down if dizziness, lightheadedness, etc, occurs. Furosemide 80 mg daily or more (or equivalent): monitor closely, consider stopping the diuretic 24 hours before starting the ACE inhibitor, or monitor for 2 hours or until the blood pressure is stable.
**1**	liraglutide	furosemide	Mild	Information is limited. Some impairment of glucose tolerance might occur. Bear in mind in case of an unexpected response to diabetic control.
**1**	Gliclazide	amiodarone	Moderate	Concurrent use should be monitored and the dose of the sulfonylurea reduced if necessary. Patients should be warned about the potential risk of hypoglycaemia.
**1**	apixaban	Aspirin	Moderate	The UK manufacturer of apixaban advises caution on concurrent use. Monitor for signs of excessive bleeding.
**1**	empagliflozin	spironolactone	Moderate	Monitor concurrent use for excessive fluid and electrolyte loss, and hypotension.
**1**	sacubitril	atorvastatin	Moderate	Be alert for an increase in statin adverse effects, and reduce the statin dose if necessary. Advise patients to report any unexplained muscle pain, tenderness, or weakness.
**1**	allopurinol	furosemide	Moderate	Use with caution, especially in patients with renal impairment. Be alert for an increase in adverse effects of allopurinol or symptoms of gout.
**1**	allopurinol	Gliclazide	Severe	The general significance of this interaction is unclear, but bear it in mind in case of an unexpected response to treatment.
**1**	gliclazide	Bisoprolol	Severe	Serious hypoglycaemic episodes are rare. Patients newly started on a beta blocker should be warned about the possible absence of hypoglycaemic warning symptoms. If diabetic control is disturbed, adjust the antidiabetic dose.
**1**	amlodipine	furosemide	Mild	No action needed unless the blood-pressure lowering effects are excessive.
**1**	Gliclazide	furosemide	Mild	Information is limited. Some impairment of glucose tolerance might occur. Bear in mind in case of an unexpected response to diabetic control.
